# Empathy as an Essential Skill of Interprofessional Collaboration in Healthcare: A Narrative Review

**DOI:** 10.3390/healthcare14060805

**Published:** 2026-03-21

**Authors:** Aikaterini Papachristou, Sofia Koukouli, Michael Rovithis, Martha Kelesi, Maria Moudatsou, Areti Stavropoulou

**Affiliations:** 1 Department of Nursing, Faculty of Health and Care Sciences, University of West Attica, Ag. Spyridonos Str., 12243 Athens, Greece; mkel@uniwa.gr (M.K.); astavropoulou@uniwa.gr (A.S.); 2 Department of Social Work, Faculty of Health Sciences, Hellenic Mediterranean University, Gianni Kornarou, Estavromenos 1, 71410 Heraklion, Greece; koukouli@hmu.gr (S.K.); moudatsoum@hmu.gr (M.M.); 3 Department of Business Administration and Tourism, School of Management and Economics Sciences, Hellenic Mediterranean University, Gianni Kornarou, Estavromenos 1, 71410 Heraklion, Greece; rovithis@hmu.gr

**Keywords:** interprofessional collaboration, empathy, IPEC competencies, ethics, communication, teamwork

## Abstract

**Background/Objectives**: Despite growing recognition of empathy as a cornerstone of high-quality care, its role within interprofessional collaboration remains underexplored. While the Interprofessional Education Collaborative explicitly references empathy only under the Values and Ethics domain, emerging evidence suggests its potential relevance across all four core competencies. This study aimed to explore the influence of empathy on each of the Interprofessional Education Collaborative core competencies and to highlight its role in the contemporary interprofessional healthcare environment. **Methods**: A narrative literature review was conducted to identify articles published in English between 2014 and 2025, through searches of PubMed and Scopus. The sub-competency statements of the Interprofessional Education Collaborative framework were used to guide keyword selection and map concepts that empathy may influence. **Results**: Seventy-two articles were included in this narrative review. According to the literature, evidence suggests that empathy may support humanitarian values and ethical decision making (Values and Ethics), but the mechanisms underlying this remain to be considered. Empathy has also been discussed in relation to therapeutic and team communication (Communication), as well as to processes such as conflict resolution, supportive leadership, team cohesion, and staff well-being (Teams and Teamwork). The evidence regarding the Roles and Responsibilities domain remains relatively limited, preventing definitive conclusions about the potential influence of empathy in this domain. A clear distinction emerges between clinical and interprofessional empathy, with evidence suggesting that both are essential for collaborative practice. **Conclusions**: Empathy appears to be linked with several domains of interprofessional collaboration and may represent an important relational component in collaborative healthcare practice. However, the influence of empathy may depend on structural and organizational conditions and may vary across different interprofessional healthcare contexts. These findings offer a conceptual bridge between empathy and interprofessional collaboration, providing actionable insights for educators, leaders, and policymakers involved in healthcare training.

## 1. Introduction

Since ancient times, Hippocrates, the father of modern medicine, emphasized the need for a deeper understanding of the patient’s experiences, needs, and feelings, laying the foundation for person-centered care.

Observing patients’ symptoms and applying an empathetic approach remain relevant and valuable, particularly in modern healthcare systems that demand coordinated interprofessional collaboration.

In recent years, researchers’ interest has strongly focused on empathy as a guarantor of high-quality patient care. Researchers seek to define and analyze the multidimensional nature of empathy to understand its contribution better and to suggest development strategies in healthcare systems [1,2,3,4,5,6,7,8]. The concept remains complex. While clinical empathy is well-established in both the literature and educational interventions, the recently introduced term of “interprofessional empathy” remains unexplored and underemphasized.

The term empathy derives from the Ancient Greek word “empátheia” [en—(“in”) and pathos (“feeling” or “experience”)], which originally denoted the presence of strong feeling or passion. Although the term has been transformed over the centuries and is attributed to modern Greek with a negative connotation—hostility, prejudice—it consolidates the linguistic connection of the word. Through its philosophical adoption in German as “Einfühlung” (feeling into), and its subsequent introduction into English as “empathy” by Edward Titchener, the term acquired its modern psychological meaning.

Davis defined empathy as a response to the experiences of others, integrating both cognitive and emotional components, such as perspective taking, empathic concern, personal distress, and fantasy [9]. In his organizational model, Davis maintains that empathy encompasses “interpersonal outcomes”, where an individual’s cognitive and affective reactions translate into behaviors that enhance the formation of social bonds and the quality of interpersonal relationships [10]. More recently, Håkansson Eklund and Summer Meranius have highlighted a clear distinction between self and other as a critical aspect of understanding, feeling, and sharing another’s emotions, a boundary essential in clinical settings [11]. As highlighted by Wei et al., effective collaboration in healthcare relies on building strong interpersonal relationships [12], a process to which empathy has been shown to contribute.

Interprofessional collaboration (IPC) is “the process by which different health and social care professional groups work together to impact care positively” [13] (p. 7). According to the World Health Organization, “Collaborative practice happens when multiple health workers from different professional backgrounds work together with patients, families, carers and communities to deliver the highest quality of care” [14] (p. 7). In modern healthcare, a supportive work environment and targeted strategies can strengthen IPC, improving job satisfaction and patient safety outcomes [15,16].

The Interprofessional Education Collaborative (IPEC) has established core competencies to prepare healthcare professionals to work together effectively, ensuring that patient-centered care is prioritized [17]. The IPEC framework consists of the following four main competencies: (1) Values and Ethics, (2) Roles and Responsibilities, (3) Communication, and (4) Teams and Teamwork. Furthermore, thirty-three sub-competency statements have been refined to broaden their applicability and articulate the knowledge, skills, and attitudes required for effective collaboration. The term “empathy” appears under the first competency area, “Values and Ethics”, yet we hypothesized that it might be a critical component to the rest of the core competencies. By recognizing the connections between empathy and core interprofessional competencies, healthcare educators and leaders can implement targeted strategies to cultivate effective IPC within their teams.

Although empathy is a widely debated topic and there is substantial evidence on how it affects patient care, less attention has been paid to how empathy interacts with the core competencies that compose IPC, such as Values/Ethics, Roles and Responsibilities, Communication, and Teams/Teamwork. This study synthesizes the existing literature to map the potential influence of empathy on interprofessional competencies within healthcare settings. By presenting the impact of empathy across the IPEC core competencies, this study strengthens the empirical basis for team leaders, educators, and curriculum designers to support the development of more effective educational interventions. It also aims to provide useful insights for team leaders, educators, and curriculum designers to support the development of more effective educational interventions.

## 2. Materials and Methods

### 2.1. Aim and Research Question

This narrative review aims to explore the influence of empathy on each of the IPEC core competencies and to highlight its role in the contemporary interprofessional healthcare environment.

Based on this aim, the following question was developed:

How does empathy influence each of the IPEC core competencies in the context of interprofessional collaboration in a healthcare environment?

### 2.2. Study Design

This study adopts a narrative review design as the most appropriate for connecting the complex nature of empathy with the multidimensional structure of interprofessional collaboration. The narrative review design allows synthesis of diverse types of evidence into a comprehensive and critical examination of the current literature. This study presents data from both theoretical and empirical studies, addressing the conceptual nature of the research question while avoiding the limitations of a rigid methodological framework, as seen in systematic reviews.

This approach allowed for a flexible yet non-arbitrary understanding of empathy in the interprofessional healthcare context. The Scale for the Assessment of Narrative Review Articles (SANRA) [18] was employed to ensure transparency and quality in the review process. SANRA criteria guided the organization and data synthesis of the present study.

### 2.3. Search Strategy

A literature search was conducted in the PubMed and Scopus databases, focusing on articles published between 2014 and 2025 and written in English. This time frame was selected to capture the literature published following the initial introduction of the Interprofessional Education Collaborative (IPEC) competency framework in 2011.

The IPEC sub-competency statements, as described in the IPEC framework [17], were analyzed to inform the identification of concepts related to empathy.

Keywords were derived from relevant concepts of empathy and incorporated into the search strategy using Boolean operators. Literature terms such as “empathy,” “ethics,” “values,” “communication,” “teamwork,” and related terms were meticulously examined to meet the research objective. Τhe term “empathy” was the main keyword, combined with either “clinical” OR “interprofessional”, and intersected with IPEC-related constructs. Additionally, the terms “healthcare”, health professions”, or “nurses” were included to ensure relevance to the healthcare context.

Suggested related articles and reference lists of key studies were hand-searched to identify additional relevant publications.

### 2.4. Eligibility Criteria and Selection Process

Titles and abstracts were screened for relevance. Full-text articles were reviewed for eligibility. The included studies were selected according to eligibility criteria.

Inclusion criteria were:

- Peer-reviewed articles published between 2014 and 2025;
- Written in English;
- Full text available;
- Empirically or conceptually examining empathy concerning one or more IPEC competencies.

Exclusion criteria were:

- Articles that exclusively focus on compassion without referencing empathy;
- Non-healthcare-related studies or non-applicable results in healthcare;
- Conference papers, unpublished, full text not available, study protocols, and gray literature.

Ambiguous cases were resolved by consensus between two reviewers.

### 2.5. Data Extraction and Synthesis of Findings

In alignment with this study’s objective to map the existing literature, findings were synthesized thematically to highlight patterns of convergence and divergence across national contexts and healthcare settings. In an initial stage, the process involved careful examination of our data and identification of specific findings or concepts related to empathy and interprofessional competencies. Recurring concepts and key findings were then highlighted and grouped into sub-categories based on their relationship to empathy and the four IPEC competencies. This framework was utilized to organize and present the synthesis of our findings, and in parallel to ensure a well-documented mapping of the relevant literature.

The findings were grouped into eight sub-categories corresponding to the four IPEC core competencies. The final synthesis highlighted the sub-dimensions in which empathy influences each IPEC core competency (Table 1). The data and interpretations were independently reviewed by two authors, with discrepancies resolved through discussion.

## 3. Results

### 3.1. Values and Ethics

#### 3.1.1. Shared Values

Empathy has been linked to nurses’ professional values [19,20] and, in some contexts, mediates their relationship with professional quality of life [21]. Empathy underpins core ethical principles in healthcare, including dignity, privacy, autonomy, and trust. Empathy is a parameter that ensures the patient’s dignity, by respecting their privacy [22], establishing trust in the physician–patient relationship [22,23,24], and providing a peaceful environment of care [25]. Empathy may also support patient autonomy by strengthening trust and encouraging patients to be involved in decision making of their care [23]. Furthermore, researchers observed that empathy enhances medical students’ attitudes towards professionalism, which is used as an “umbrella term” for critical values and qualities in the provision of high-quality care [26].

González-Serna highlighted the significant role of empathy in fostering prosocial behavior and in shaping the perception and application of professional ethical values [27]. Empathy has been linked to prosocial behavior, since it motivates individuals to make appropriate decisions through affective responses to others’ suffering [28]. Nevertheless, empathy does not automatically translate into prosocial action, as emotional distress and overload may lead to avoidance rather than helping behavior [29]. Helping behaviors, which are facilitated through perspective taking and emotional connection, depends on emotion regulation processes [30].

Altruism and empathy are closely related phenomena [31], particularly in terms of the motivation to care, the willingness to help [32], and the prioritization of patients’ interests through selflessness [33]. Health professionals commonly describe empathy as an altruistic obligation or endeavor [7], often expressed through key phrases such as “treating others the way you want to be treated yourself” and “how would I feel if I were in their place”. However, relevant research suggests that ethical motivations such as altruism may be experienced ambiguously in clinical practice and shaped by professional expectations and organizational contexts [31].

Furthermore, different dimensions of empathy exhibit divergent relationships with moral decision making; it is cognitive—not affective—empathy, that actively motivates the promotion of justice for others [34]. In addition, the high value placed on patients’ advocacy—characterized by the intention to combat injustice and inequality—is implicitly rooted in empathy [35].

Particularly in racial and culturally diverse clinical settings, empathy is increasingly recognized as an important component in advancing health equity and fostering respect for diversity. Higher levels of interpersonal and social empathy have been linked to more positive attitudes toward racial diversity and lower levels of prejudice [36]. By cultivating trust, supporting equitable interactions, and promoting patient engagement [37], empathy can help reduce racial disparities in healthcare. Improved levels of empathy following a simulation-based training program have been associated with shaping individual attitudes and increasing awareness of racial bias [38]. It also plays a central role in addressing social stigma, as studies have shown that empathy-enhancing experiences can improve healthcare professionals’ attitudes towards people living in poverty [39,40]. Meanwhile, greater empathic understanding helps combat stigma against people with mental illnesses [41]. Additionally, training techniques such as simulations [40], and health disparities programs [42] have been shown to strengthen students’ empathy and reduce bias toward vulnerable groups, hence fostering more respectful and equitable care. Although empathy-focused educational interventions have been shown to increase awareness of social inequalities, evidence regarding their long-term impact on healthcare disparities remains limited.

#### 3.1.2. Ethical Conduct

Concerning ethical conduct, empathy serves as a significant mediating factor in the relationship between mindfulness and moral sensitivity [43]. Several studies have shown a correlation between moral sensitivity and empathy. Suazo et al. found that cognitive empathy enhances the humanization of care and mediates the relationship between moral sensitivity and prosocial behavior in nursing practice [44].

Furthermore, recent research conducted by Chen et al. demonstrated that empathy not only exhibits a positive correlation with caring behaviors but also with moral sensitivity [45]. This finding highlights the mediating role of empathy among nurses, suggesting that it may be a crucial determinant of both moral sensitivity and the performance of caring behaviors in clinical settings. Liu et al. identified a direct effect of empathy on moral sensitivity, emphasizing the role of emotional intelligence as a mediating factor in this relationship [46].

Empathy directly influences ethical sensitivity positively. Yuguero et al. demonstrated a significant association among moral development, ethical sensitivity, and elevated levels of empathy [47]. Additionally, research by Sharifnia et al. indicates that individuals with higher empathy levels tend to exhibit greater ethical sensitivity [48]. This enhanced ethical sensitivity may serve to alleviate the adverse effects of emotional exhaustion, particularly in high-pressure environments. Furthermore, Pohling et al. noted that affective empathy exerts distinct effects on ethical competence, with values mediating in this relationship [49].

Integrating empathy into the ethical decision-making process is crucial for navigating the moral complexities of healthcare. Furthermore, enhancing empathy and professional values may improve nurses’ ethical decision-making capabilities [50].

### 3.2. Roles and Responsibilities

#### Understanding of Roles and Professional Identity

Higher levels of empathy foster a deeper comprehension of professional roles and positively alter attitudes of other health professionals in interprofessional educational programs. Empathy is a crucial mechanism for connecting with colleagues at both emotional and cognitive levels, especially through the development of perspective taking and empathetic concern [51].

Valenziano et al. observed that interprofessional empathy facilitates recognition of others’ professional challenges, responsibilities, and skills [52]. Also, Chen et al. found that students participating in IPE simulations reported improved understanding of one another’s roles and greater empathy for their colleagues [53].

A positive correlation was found between empathy and the formation of professional identity [54]. This result is also supported by Rachakonda et al., who found that empathy, among other skills, significantly contributes to the formation of professional identity in health professionals [55]. Targeted emotional intelligence training is promising for developing empathy and professional identity in young health professionals, equipping them with the skills to provide humanistic care and fulfill their professional role [56]. Similarly, early exposure to the clinical learning environment seems important in fostering empathy and developing professional identity [56].

### 3.3. Communication

#### 3.3.1. Effective Communication with Patients and Families

Empathetic care is based on effective communication [57]. Therapeutic communication and empathy are inextricably linked. Most measurement tools for therapeutic communication—such as the Global Interprofessional Therapeutic Communication Scale (GITCS) [58] and the Therapeutic Communication Scale [59]—explicitly include empathy as a key component. Similarly, in the scale developed by Ghiyasvandian et al., empathy appears as a distinct subscale, emphasizing emotional responsiveness and professionals’ ability to view situations from the patient’s perspective [60]. Moreover, empathy map exercises in clinical education incorporate an empathy dimension to strengthen patient-centered care [61]. Empathy in communication promotes a common sense of humanity and better understanding of patients’ perspectives, leading to the recognition of the patients’ real needs, contributing decisively to developing a therapeutic relationship [61]. Studies have shown that empathic communication enhances overall patient satisfaction [62,63]. For nurses in particular, active listening and empathy are among the most essential communication skills of the therapeutic relationship [64], significantly influencing patient satisfaction within the therapeutic interaction [62]. Nursing students with high interpersonal communication skills demonstrate higher levels of empathy towards patients [65]. In addition, an empathetic approach helps nurses communicate better and manage serious illness conversations [66], and difficult situations characterized by patients’ disrespect and rudeness [22]. Patients and family perceive empathy through how team members communicate in multiple ways, such as authentic, effortless verbal communication, therapeutic touch, and non-verbal communication [67].

#### 3.3.2. Team Members’ Communication

In recent years, a new term “interprofessional empathy”—referring to the empathy within members of an interprofessional team—has been introduced, separated from the concept of clinical empathy.

The direct link between communication and empathy creates a two-way relationship that influences these two skills in the interprofessional environment. Communication skills training resulted in the development of empathy [68,69]. Empathy has been recognized as a mediating factor between communication competence and interpersonal competence [70]. Communication competence promotes high team performance [67]. Communication training programs, such as relationship-centered workshops, that include the empathy dimension, showed significant improvements in interpersonal interactions, thereby enhancing professional networks [71]. Additionally, training in non-violent communication has been effective in increasing empathy levels in medical students [72] and nursing students [73], while also leading to significant outcomes in trust, open dialog, and common language that fosters IPC [74].

Communication is an essential factor in IPC, fostering the development of empathetic relationships, with dialogic communication serving as a foundation in these interactions [7]. Empathy as a form of verbal communication is a prerequisite of mutual understanding, perspective taking, and a collective spirit within interprofessional teams [7].

Another notable observation came from André et al., who conclude that empathy and “concern for others” are significantly reduced during communication under stressful situations, underlining the importance of maintaining a healthy working environment in cultivating empathetic communication between healthcare providers [75].

### 3.4. Teams and Teamwork

#### 3.4.1. Empathetic Leadership and Supportive Workplace

Leadership is not only a managerial function but also a cultural force that shapes interprofessional dynamics and influences the overall quality of patient care. Empathetic leadership fosters a fair, psychologically safe, and supportive workplace, which is essential for promoting team cohesion and professional well-being. According to Kang et al., interprofessional empathy can contribute to health professionals’ well-being, which in turn enhances the delivery of empathetic care [76]. Fuentes et al. found that empathy serves as a mediation factor between mindfulness and work engagement in nurses, providing significant benefits for members’ well-being, their team’s work commitment, and better patient outcomes [77]. Similarly, leadership behaviors that foster psychological safety enable interprofessional teams to work more cohesively [78]. Empirical findings indicate that empathetic leadership was associated with higher employee resilience, though it is not significantly related to turnover intention. Because this study was conducted during the COVID-19 pandemic, these findings should be interpreted with caution and may lack broader generalizability [79].

Leadership has a critical responsibility to implement organizational reforms that ensure good working conditions, openness, and psychological safety, and cultivate an environment conducive to the development of empathy and teamwork. Healthcare leaders and organizations are encouraged to align their strategies with these findings to improve staff engagement and patient outcomes. Without empathetic leadership and institutional support, even well-designed educational programs may fail in achieving lasting changes or improved patient care.

#### 3.4.2. Team Members’ Relationships

Empathy strengthens healthcare team members’ relationships by fostering respect and understanding [80]. Berduzco-Torres et al. observed that developed empathy contributes to a higher capacity for teamwork and forms more stable working relationships among medical and nursing students, regardless of their professional discipline [81]. Similarly, Zelenski et al. observed a positive effect in interprofessional relationships following an empathy-targeted course that employed theater techniques [82]. Hasson et al. developed a performance-art intervention to promote the belief that “empathy is unlimited” and found that it increased outgroup empathy, supported prosocial actions, and reduced intergroup bias in face-to-face interactions [83].

Researchers observed a strong positive correlation between empathy and teamwork [81,84,85]. Hojat et al. suggested that integrative patient care can promote empathetic patient interactions and foster teamwork by enhancing interpersonal skills and establishing a common understanding of patients’ needs [84]. Shared experiences and conscious interactions develop rapport, which is crucial in nurturing interprofessional empathy [7,52]. Collegiality and approachability are fostered by empathy-based professional relationships, which are beneficial not only in the clinical setting but also when they are extended outside of it [52]. Adamson et al. further emphasize that empathy not only enhances intergroup relations but also improves teamwork dynamics, leading to more effective IPC [7].

#### 3.4.3. Critical Teamwork Skills

Interprofessional empathy does not refer only to harmonious behaviors. It also plays an important role in conflict resolution by helping members recognize and negotiate differences [7]. A decrease in empathy due to adverse working conditions and stressful situations is associated with higher stress-driven conflicts among team members [86]. Considering Thomas Kilmann’s conflict management model, Hastings et al. concluded that pharmacy students’ higher scores on the competing mode were associated with lower attitudes toward empathy [87]. On the contrary, students with higher attitudes towards empathy choose the accommodating mode to manage conflicts.

Another essential skill for the effectiveness of a healthcare team is the ability to constructively resolve emerging problems. Higher levels of cognitive empathy are associated with higher problem-solving scores among operating room nurses [86].

Healthcare work environments are highly demanding, highlighting another important aspect of individual and group dynamics, namely resilience. Research has shown that resilience and empathy are positively correlated [88,89], while a negative correlation between empathy and burnout has been observed [90].

## 4. Discussion

This review aimed to map the existing literature and to explore the influence of empathy on interprofessional competencies within healthcare settings. Potential connections were identified between empathy—both clinical and interprofessional—and the IPEC core competencies. A central theme emerging from the synthesis is that empathy may play a motivating, supporting, and reinforcing role in several domains of the IPEC framework; however, the strength and mechanisms of these relationships remain unevenly documented and require further empirical investigation.

According to the literature, evidence suggests that empathy may support humanitarian values and ethical decision making (Values and Ethics), but mechanisms have not yet been adequately examined. Empathy has also been discussed in relation to therapeutic and team communication (Communication), as well as to processes such as conflict resolution, supportive leadership, team cohesion, and staff well-being (Teams and Teamwork). The evidence concerning the Roles and Responsibilities domain remains relatively limited, which prevents definitive conclusions regarding the potential influence of empathy in this domain. The evidence appears stronger for the communication domain and moderate for Values/Ethics and Teams/Teamwork. However, several studies suggest that the influence of empathy on team processes may depend on organizational culture, hierarchical structures, and contextual factors in healthcare organizations, all of which require careful consideration.

This study suggests that the adoption of empathy within the team, towards both professionals and patients, could be considered an essential skill for interprofessional collaborative practice. However, developing empathy requires effort, through conscious interactions, group experiences inside and outside the work environment, and targeted educational interventions.

Previous research supports our findings, reporting significant links between empathy, teamwork, and IPC [84] and highlighting its role in successful collaborative efforts within healthcare teams. Our consideration of empathy as an important component for interprofessional collaborative practice agrees with Adamson et al., who emphasize the importance of empathy, characterizing it as a precursor to IPC [7]. Moreover, several studies support the idea that the development of empathy is not accidental [91], focusing on team members’ interactions, using similar wording, such as “conscious interactions” [7], “intentional and genuine team experiences” [91], “rapport” and “shared experiences” [52].

In their systematic review, Schot et al. identified that health professionals contribute to IPC in three core ways: “by bridging multiple types of gaps, negotiating overlaps in roles and tasks, and by creating new spaces” [92] (pp. 338–339). This notion of “bridging gaps” aligns strongly with the metaphor used by Adamson et al., who characterize perspective taking—an integral component of interprofessional empathy, as analogous to “building a bridge” across different viewpoints and professional identities [7]. Consequently, synthesizing these insights, one might argue that empathy may serve as a critical component in IPC, as it empowers professionals to bridge cognitive, emotional, and systemic gaps that naturally arise when diverse disciplines work together.

Ιn recent studies, there is an attempt to distinguish the general term of empathy into “clinical” [4,6,93,94] when referring to the healthcare professional–patient relationship and “interprofessional” when addressed concerning interaction between health professionals [7,52,91,95,96]. Despite the significance of this distinction, we observed that both are equally essential for collaborative practice. Often being conflated, each serves distinct but complementary functions within the framework of interprofessional collaboration in healthcare. A mutual understanding of “clinical” empathy creates a value system and a shared framework for decoding patients’ needs, fostering teamwork, and a sense of common purpose. On the other hand, “interprofessional” empathy involves accepting another viewpoint among team members, promoting interprofessional communication, nurturing team spirit, and facilitating conflict resolution, thereby facilitating IPC. The development of empathy within interprofessional teams should include both dimensions of “clinical” and “interprofessional” to bring about the desired changes and to address the challenges that arise in pursuing IPC. Emphasis should be placed on cultivating interprofessional empathy, which, as a recently introduced concept, is significantly under-represented in training and research compared to clinical empathy.

Despite its significance, empathy remains a common weakness in healthcare collaboration, particularly in high-stakes areas like palliative and end-of-life care [64,67]. The good news is that empathy can be enhanced by teaching [97,98]. Training programs that incorporate communication skills and experiential learning in highly demanding empathic environments have shown positive results in developing this skill [68,99].

Interprofessional education (IPE) is a key strategy for interprofessional collaborative practice. Research shows that empathy enhances readiness for interprofessional learning [85], and IPE simulations improve communication, teamwork, and mutual understanding across disciplines [53,80]. Chua et al. confirm that IPE boosts empathy by fostering more empathic responses toward both patients and peers [96]. Similarly, McNulty and Politis highlight the need to integrate empathy and emotional intelligence into team-based healthcare education to promote person-centered care [100].

While the effectiveness of IPE is well-supported and recognized as a suitable approach to fostering collaboration in healthcare, its implementation is often restricted to academic settings due to practical considerations within clinical environments, such as shift coverage and workload. There is insufficient research to determine how educational interventions within a single profession (single-professional or uni-professional education) influence IPC. Chen et al. found that both educational approaches are effective in enhancing team behavior performance [53]. Similarly, Park et al. found that both educational approaches yield the same outcomes in the context of IPC [101]. However, IPE appears more advantageous because of direct interaction among different professionals. Nevertheless, the single-professional approach remains a viable, efficient alternative, with the primary advantage of not being subject to the limitations of the interprofessional approach. Future research is encouraged to further explore empathy-focused interventions, both single-professional and interprofessional, and their impact on IPC. Given interprofessional empathy’s central role in teamwork and communication, empathy-based training in a particular discipline and its outcomes in IPC remain important and timely areas for future research.

However, empathy is highlighted as an essential skill for IPC; the literature has emphasized that it is not always beneficial. Several recent studies criticize empathy, especially affective empathy, for bias and risks, leading to short-sighted moral reasoning [102]; emotional exhaustion; reactive aggression [103]; mental health risks such as anxiety, depression, and burnout [104,105]; unequal care; and undermining patients’ autonomy [106]. Furthermore, researchers underpin the complex relationship between empathy and morality, noting that empathy is a multifaceted construct whose different components may shape moral reasoning in divergent and, at times, biased ways [107]. Cultural variability and contextual factors may further influence how empathic behaviors are expressed and interpreted in healthcare environments [108]. Conceptual ambiguity between empathy, sympathy and compassion has been noted in the literature, which complicates both measurement and practical application in clinical settings [109]. These findings suggest that empathy should be understood as a complex and multifaceted construct whose influence on clinical practice and interprofessional collaboration may vary across settings.

These findings underscore critical limitations that educators and leaders should recognize to design more effective empathy-based interventions that support interprofessional collaboration (IPC). From an educational standpoint, the findings suggest that initiatives seeking to enhance interprofessional collaboration may benefit from incorporating empathy-related elements, such as communication skills training, experiential or simulation-based learning, emotional intelligence development, emotion regulation training, and reflective practices within interprofessional education programs. Educational interventions addressing interprofessional collaboration may also consider both dimensions of empathy, clinical empathy and interprofessional empathy, as potentially relevant components for supporting collaborative practice in healthcare settings. Educational interventions should prioritize increasing awareness of personal and professional biases. Through well-designed training, empathy can efficiently support interprofessional practice. However, without such structured guidance, it may negatively impact team dynamics, staff’s mental health, and ethical decision making.

Maintaining empathic practice requires more than individual training; however, Nembhard et al. [110] identified a significant lack of organizational-level interventions. A broader organizational shift, supported by leadership and prioritizing staff well-being, is urgently needed [7,111]. Such practice relies on supportive leadership, a shared vision, and collaborative healthcare environments [112,113]. To achieve enduring results in IPC, training programs focused on the development of empathy must be supported by organizational changes that foster open communication, trust, and employee well-being.

### 4.1. Conceptual Implications and Future Directions

This review provides a conceptual synthesis of the literature on empathy within the framework of the Interprofessional Education Collaborative (IPEC) competencies. By mapping findings across the four IPEC domains (Values and Ethics, Roles and Responsibilities, Interprofessional Communication, and Teams and Teamwork), it offers an overview of how empathy has been discussed in relation to different aspects of interprofessional collaboration in healthcare.

This review may serve as a preliminary step toward the development of a conceptual framework that integrates empathy within interprofessional competency models. Furthermore, from an educational perspective, this synthesis may inform interprofessional education (IPE) by highlighting how empathy relates to different competency domains. This understanding may support the design of educational activities that integrate empathy with communication skills, teamwork, and collaborative problem solving.

While emerging evidence suggests that empathy may contribute to interprofessional collaboration under specific conditions, the existing literature remains insufficient in drawing definitive conclusions about the mechanisms through which this influence occurs. Future reviews may benefit from including additional databases and multilingual sources to further broaden the scope of evidence. Future research could further examine how empathy interacts with specific interprofessional competencies and how these relationships influence collaborative practice in healthcare settings. Empirical studies using validated measures of empathy could help clarify the strength and nature of these associations. Moreover, although several instruments have been developed to measure empathy in healthcare environments, to our knowledge, there is currently no validated tool specifically designed to assess interprofessional empathy within collaborative healthcare settings. Longitudinal and intervention studies may also provide valuable insights into how educational programs influence empathy development and interprofessional competencies over time. Future research could further explore the psychosocial dimensions of empathy in healthcare teams, particularly the interactions between empathy, professional well-being, resilience, and organizational climate, as these factors may significantly influence interprofessional collaboration. Additionally, exploring organizational and contextual factors, such as team climate, professional culture, and workplace support, may contribute to a more comprehensive understanding of how empathy operates within interprofessional teams.

### 4.2. Limitations

This study acknowledges several limitations.

The subject matter is broad, encompassing numerous dimensions of how empathy relates to core competencies and IPC. This wide scope makes it challenging to explore every relevant aspect thoroughly. Given the complexity of the subject, some key elements may have been overlooked. To address this limitation, the IPEC sub-competency statements served as a guide to identify relevant keywords, narrowing the focus to the most pertinent evidence. This approach ensures that the most critical aspects and essential ideas are adequately explored within the available scope. This study followed a narrative review approach to explore the broad scope of empathy within interprofessional collaboration in healthcare; however, the lack of quality appraisal and the heterogeneity of the included studies do not allow direct comparison of findings. Also, the use of only two databases for the literature search and the restriction to English-language publications may have resulted in part of the relevant literature remaining outside the scope of this review.

Empathy in the included studies was commonly assessed using self-report instruments, such as the Jefferson Scale of Empathy and the Interpersonal Reactivity Index, which evaluated the cognitive and affective dimensions of empathy. Although such tools provide useful insights into empathic attitudes and perceptions, the reliance on self-reported measures may introduce response bias and limit the comparability of findings across studies.

Evidence supporting the relationship between empathy and the “Roles and Responsibilities” domain is limited. This limitation may have arisen due to the absence of standardized terminology, the small number of studies in the field, and the unproductive keyword searches yielding few eligible articles. This limitation creates an imbalance in the results of this study compared to other domains and may affect the ability to draw fully comprehensive conclusions about the overall influence of empathy in IPC. To mitigate this limitation, the most recent and relevant articles have been included. Although the evidence base is limited, it is sufficient to support the analysis.

Another limitation of this study is based on the potential confusion between the terms “empathy” and “compassion”. These two terms are often used interchangeably in research, leading to a blurring or misunderstanding of their distinct meanings. This overlap of concepts is often a challenge for studies that examine empathy-related contexts, as it is possible to include compassion-related outcomes and vice versa. To address this issue, we extracted articles that include “compassion” and selected evidence that directly referred to “empathy”. By this inclusion–exclusion approach, the study was able to maintain clarity and precision in its analysis.

## 5. Conclusions

In summary, empathy appears to be linked with several domains of interprofessional collaboration and may represent an important relational component in collaborative healthcare practice. However, the strength of the evidence was not consistent across all competencies within the IPEC framework, with more limited indications in domains such as Roles and Responsibilities. Empathy is a multidimensional construct whose influence may vary depending on contextual, organizational, and team-related factors within healthcare settings. Therefore, the mechanisms through which empathy may influence collaborative practice should be interpreted with caution and in relation to the specific characteristics of each professional environment. Further empirical research is needed to better understand how empathy influences interprofessional collaboration and team functioning in healthcare contexts.

Incorporating empathy-targeted interventions into training programs may enhance interprofessional collaboration (IPC). This approach may also foster greater attention to the measurement and development of interprofessional empathy, a concept that remains under-explored in the context of collaborative healthcare practice. Furthermore, future research is encouraged to delve deeper into empathy-focused interventions, both single-professional and interprofessional, and their impact on collaborative practice. The integration of empathy into educational programs and curricula is crucial for preparing healthcare professionals and students to collaborate effectively and provide high-quality patient-centered care. To achieve sustainable change, empathy training must be embedded within organizational culture and supported by system-wide strategies that promote IPC.

## Figures and Tables

**Table 1:** Empathy-related findings mapped to IPEC core competencies.

IPEC Competency Dimensions	Sub-Dimensions	Key Concepts and Findings Related to Empathy
Values and ethics	Shared values	Dignity, privacy, autonomy, trust
		Prosocial behavior
		Professionalism
		Altruism
		Justice
		Health equity, inclusion
	Ethical conduct	Moral sensitivity
		Ethical sensitivity
		Ethical decision making
Roles and responsibilities	Understanding of roles and professional identity	Perspective taking of other professionals
		Improvement in understanding roles
		Recognition of the contributions and challenges faced by healthcare professionals from other disciplines
		Formation of professional identity
Communication	Effective communication with patients and families	Therapeutic communication
		Patient support, patient-centered care
		Sharing, understanding, trust
		Overall patient satisfaction
		Management of difficult conversations and situations
	Team members’ communication	High team performance
		Interpersonal competence
		Trust, open dialog, common language
		Dialogic communication
		Mutual understanding, perspective taking, collective spirit
Teams/Teamwork	Empathetic leadership and supportive workplace	Staff’s well-being
		Emotional support
		Work engagement and commitment
		Psychological safety
	Team members’ relationships	Respect, mutual understanding
		Stable working relationships
		Reduction in intergroup bias
		Integrative patient care
		Collegiality, approachability
	Critical teamwork skills	Conflict resolution and reconciliation
		Problem solving
		Resilience

## Data Availability

Data sharing is not applicable. No new data were created or analyzed in this study.

## Abbreviations

The following abbreviations are used in this manuscript:

GITCS	Global Interprofessional Therapeutic Communication Scale
IPC	Interprofessional collaboration
IPEC	Interprofessional Education Collaborative
SANRA	Scale for the Assessment of Narrative Review Articles
